# Efficient link prediction in the protein–protein interaction network using topological information in a generative adversarial network machine learning model

**DOI:** 10.1186/s12859-022-04598-x

**Published:** 2022-02-19

**Authors:** Olivér M. Balogh, Bettina Benczik, András Horváth, Mátyás Pétervári, Péter Csermely, Péter Ferdinandy, Bence Ágg

**Affiliations:** 1 Cardiometabolic and MTA-SE System Pharmacology Research Group, Department of Pharmacology and Pharmacotherapy, Semmelweis University, Nagyvárad tér 4, Budapest, 1089 Hungary; 2 Faculty of Information Technology and Bionics, Pázmány Péter Catholic University, Budapest, Hungary; 3 Pharmahungary Group, Szeged, Hungary; 4 Department of Molecular Biology, Semmelweis University, Budapest, Hungary

**Keywords:** Edge prediction, PPI prediction, Protein interaction prediction, Interactome, Conditional GAN

## Abstract

**Background:**

The investigation of possible interactions between two proteins in intracellular signaling is an expensive and laborious procedure in the wet-lab, therefore, several in silico approaches have been implemented to narrow down the candidates for future experimental validations. Reformulating the problem in the field of network theory, the set of proteins can be represented as the nodes of a network, while the interactions between them as the edges. The resulting protein–protein interaction (PPI) network enables the use of link prediction techniques in order to discover new probable connections. Therefore, here we aimed to offer a novel approach to the link prediction task in PPI networks, utilizing a generative machine learning model.

**Results:**

We created a tool that consists of two modules, the data processing framework and the machine learning model. As data processing, we used a modified breadth-first search algorithm to traverse the network and extract induced subgraphs, which served as image-like input data for our model. As machine learning, an image-to-image translation inspired conditional generative adversarial network (cGAN) model utilizing Wasserstein distance-based loss improved with gradient penalty was used, taking the combined representation from the data processing as input, and training the generator to predict the probable unknown edges in the provided induced subgraphs. Our link prediction tool was evaluated on the protein–protein interaction networks of five different species from the STRING database by calculating the area under the receiver operating characteristic, the precision-recall curves and the normalized discounted cumulative gain (AUROC, AUPRC, NDCG, respectively). Test runs yielded the averaged results of AUROC = 0.915, AUPRC = 0.176 and NDCG = 0.763 on all investigated species.

**Conclusion:**

We developed a software for the purpose of link prediction in PPI networks utilizing machine learning. The evaluation of our software serves as the first demonstration that a cGAN model, conditioned on raw topological features of the PPI network, is an applicable solution for the PPI prediction problem without requiring often unavailable molecular node attributes. The corresponding scripts are available at https://github.com/semmelweis-pharmacology/ppi_pred.

**Supplementary Information:**

The online version contains supplementary material available at 10.1186/s12859-022-04598-x.

## Background

Biological processes of the cell are mediated by various biomolecules, especially proteins and their interactions via temporary or long-term physical contacts. Therefore, the investigation of protein–protein interactions (PPIs) may lead to a better understanding of cellular functions and disease mechanisms, as well as to improvements in drug design [1–3]. The knowledgebase that describes the intricate relationships between interacting biomolecules can be represented as a network, called PPI network (also called the interactome), in which the emerging topological features may unveil previously undetected properties of biological phenomena. The PPI network is highly incomplete in terms of discovered edges, as the validation of possible interactions is commonly performed via expensive and laborious experimental methods, such as yeast two-hybrid systems [4], mass spectrometry [5] or protein microarrays [6]. Thus, several computational methods were proposed in the recent two decades, aiming to provide an in silico approach that is able to analyze the known interactions and predict the most probable unknown ones for further wet-lab investigations [7]. Traditional in silico approaches, that are still subjects of studies include for example the docking algorithms, that exploit the predicted 3-dimensional structures of proteins and rigorously simulate each possible protein–protein association in molecular assemblies, calculating the binding affinity between the participants [8, 9]. However, advances in the field of bioinformatics and data science introduced newfound approaches that transformed the PPI prediction problem into a machine learning task for already established models, such as random forest classifiers [10], support vector machines (SVMs) [11, 12], weighted sparse representation-based classifiers [13] and artificial neural network models, like stacked autoencoders [14], feed forward neural networks [15, 16] and deep convolutional networks [17]. These studies utilized various combinations of protein features, including the sequences and their transformations, handcrafted physicochemical properties, domain knowledge or even gene ontology annotations. In our work, the network representation of the interactome and the use of strictly topological features, as opposed to molecular attributes, should provide a novel perspective for the PPI prediction task via methods developed for link prediction problems.

The link prediction problem was first formalized for the purpose of investigating the dynamically changing topology in large social networks, in which information has to be extracted from a given snapshot of the network and is used to infer new connections among the members of the community in the next snapshot [18]. Methods that are viable for predicting the future evolution of dynamic networks might also be successfully applied to discover missing links in incomplete static networks [19]. At the highest level of abstraction, two distinct classes can be defined for the task: heuristic-based and learning-based approaches [20]. The calculation of heuristic information is the more traditional way of describing the features of the nodes, and consequently their similarity, which is the determining factor for the prediction. We may further divide this category, by the source of heuristics, into node neighborhood-based similarity measurements, such as common neighbors, Jaccard coefficient, Adamic/Adar score and preferential attachment, and into ensemble of all path-based heuristics, such as Katz method, hitting time, Rooted PageRank and SimRank [18, 20]. Although these algorithms provide a strong baseline of evaluation, recent studies favored the investigation of new learning-based techniques, in which node similarity is learnt through various machine learning models. The two major classes of approaches of learning can be defined yet again by the sources of node attributes [20]. Using calculated topological features, like the aforementioned heuristics themselves, the information describing the node pairs can be fed into any classifier, such as SVMs, k-nearest neighbors, decision trees or neural networks [21]. Via supervised learning, these models are trained to extract the defining features from the provided values and classify the pairs as either edge candidates or not. The more complex way of providing node information for a machine learning model is implemented via various unsupervised methods, which aim to autonomously construct feature vectors from the underlying data distribution. These latent-feature-based models include broader categories, such as tensor factorization-based methods, nonparametric models and deep learning, using neural networks [20]. Focusing on the latter, the features extracted via neural network models are either used by another classifier, like in the case of natural language processing inspired embedding algorithms [22, 23], or used by the same model in further processing, like in the case of generative models, such as restricted Boltzmann machines (RBMs) [24] and deep belief networks (stacked RBMs) [25]. To the best of our knowledge, studies regarding link prediction-based PPI network analysis, that utilize machine learning, mainly focused on the use of graph embedding techniques and simple classification models [26, 27], leaving more complex latent-feature-based generative models untested.

Recent advances in neural network architectures resulted in one of the most prominent and widely used generative model frameworks, the generative adversarial networks (GANs) [28]. Originally, GANs gained fame in the field of image generation, but later the model was successfully adapted to a variety of other uses, including recommendation systems [29], embedding [30, 31], labeled data generation [31], and the prediction of network evolution in dynamic social networks [32, 33]. GANs consists of two interacting artificial neural networks, called the generator and discriminator, that are participating in a mini-max game against each other, enforcing their opponent to improve its performance during the learning process. This is a supervised way of training an unsupervised generator model, which aims at learning to produce outputs that are indistinguishable by the discriminator and ideally by human agents as well [28]. The original GAN architecture takes random noise as its generator input, which can be complemented with samples of a prior distribution to achieve more desirable quality of the outputs. In the case of samples being provided as conditions, the discriminator part of the conditional GAN (cGAN) will be tasked to judge the transformation itself on the sample level, that maps the prior distribution into the target distribution [34]. In the case of the provided condition being similar in representation and dimensions to the target output, the work of the cGAN might be referred to as image-to-image translation [35]. Using this approach, the input random noise can be excluded entirely to enforce a direct and deterministic mapping of the initial image into its target form.

Considering the high-quality outputs of these architectures in the field of image generation and translation, our aim was to apply a modified GAN model to the link prediction problem in an incomplete graph, rather than a dynamic one. Given, that the features of a network can be represented and learnt by the GAN, similar to images, the link prediction task in PPI networks might be performed using only topological information of the interactome, eliminating the need of molecular protein attributes from external databases.

## Implementation

The implementation of our link prediction tool contains two types of frameworks: one serves the purpose of performance evaluation, generating predictions that are comparable with the expected original network, and the other serves the purpose of predicting entirely unknown links in the original network for further validation. The two frameworks require slightly differing preprocessing and machine learning steps, but they share the basic structure of the software, which consists of a preliminary network processing module and a machine learning model. The description of the implementation will focus on the scripts used for performance evaluation, as those were applied to generate the presented results.

### Preprocessing

**Fig. 1:**
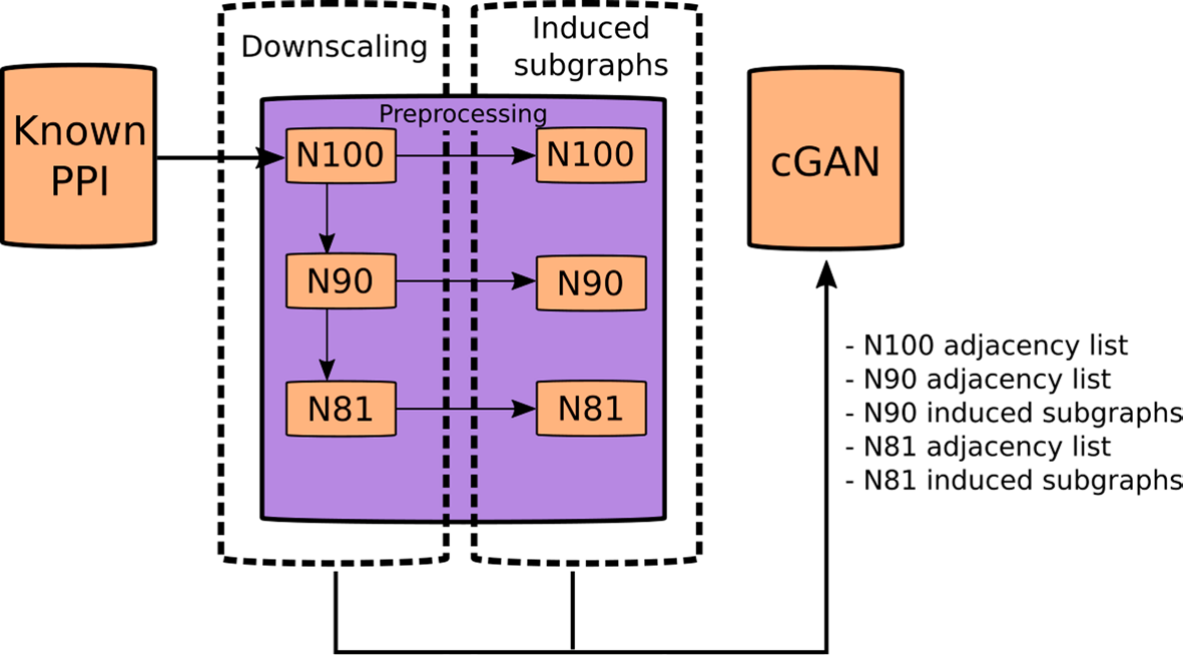
**Preprocessing of the input network.** Schematic summary of the preprocessing module, that takes in the provided protein–protein interaction (PPI) network, and produces the downscaled networks with 90% (N90) and its 90% (thus 81%, N81) of edges from the original one (N100) in the form of adjacency lists, and generates the induced subgraphs for each. These representation files are created for the original network as well but are not required in the machine learning part, resulting in the listed 5 files to be fed into the conditional generative adversarial network (cGAN) model down the line

The preprocessing module is responsible for generating the input files for the machine learning model. These files contain the various network representations of the original input PPI network, which were created in one of the two distinct steps of the preprocessing (Fig. 1), chained together via shell scripts.

### Downscaling

The downscaling step is written in Python, in the same script as the induced subgraph extraction, however its algorithm is executed first. The input network is given as an adjacency list in CSV (Custom Separated Value) format which is denoted by N100. For tenfold cross-validation, truncated networks are generated with scikit-learn K-Folds cross-validator function [36], keeping only 90% of the known edges in the new networks denoted by N90 (Fig. 1). During 10-Fold cross-validation, for each training of the machine learning model an N90 network is truncated once again via the train-test split function of scikit-learn, keeping 81% of the original edges as a new network, denoted by N81. In each downscaling operation, all resulting network components are kept, regardless of size, as the next preprocessing step will filter out the insufficient components either way. The N81-N90 network pairs are used to train the machine learning model to properly transform a given network into its more connected form. Following the training, the N90-N100 network pairs are utilized to evaluate the performance of the model.

### Induced subgraphs

Adjacency matrices of entire PPI networks would prove to be not only too large in dimensions to be processed due to memory constraints, but also too sparse to be useful due to model convergence difficulties. Thus, the N81 (for training) and the N90 (for evaluation) networks are broken down into several smaller pieces via a simple module detection method (Fig. 1). Starting from each node of the N90 and N81 networks, induced subgraphs are created, that describe their local neighborhood by discovering the connected nodes through an uninformed search. These induced subgraphs are assessed by a modified version of the classical breadth-first search algorithm [37] which is iterated over layers containing *n*_*i*_ neighbors of the nodes of the previous layer until the traversed *k* layers altogether contain less nodes than a predefined limit *l* (in our case *l* = 36):

$$s_{k} = \sum\limits_{i = 1}^{k} {n_{i} < l.}$$

**Fig. 2:**
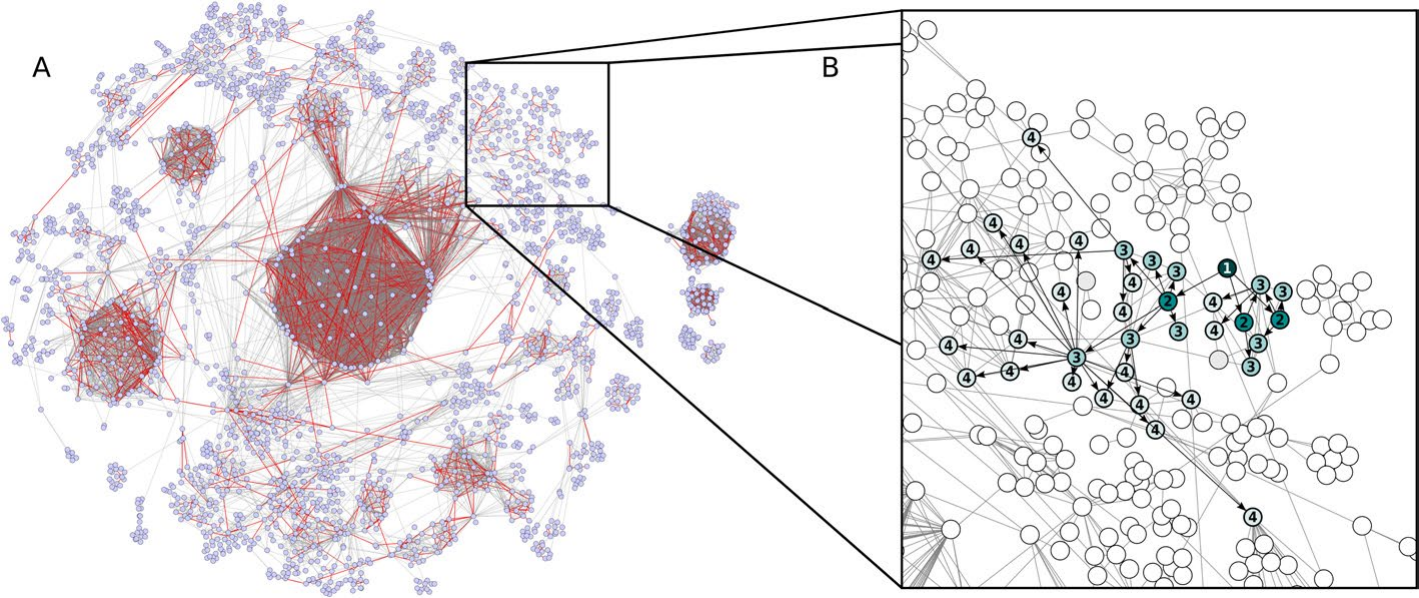
**Data preprocessing: downscaling and induced subgraph generation.** Human protein–protein interaction (PPI) network (N100) with red colored edges to be deleted in the tenfold cross-validation stage to retrieve training dataset (N90) in one example fold (**A**). The networks constructed from truncated datasets (N90) generated with tenfold cross-validation are traversed with a modified version of the classical breadth-first search (BFS) to extract equal-sized induced subgraphs which serve as input for conditional generative adversarial network (cGAN) (see Fig. 1). Example induced subgraph node color intensity and node labels represent depth level of the modified BFS. In contrast to the classical BFS, the traversal was supplemented with size specifications and on the last depth level of modified BFS, nodes were randomly selected (possible nodes on last level: gray nodes; selected nodes on last level: pale green nodes with label) (**B**). Only network components covered by modified BFS are shown in these representations (giant component and appropriately sized isolated components). See Additional file 2: Fig. S5 for high-resolution image of the representative initial full network with node annotation

At the last step of this modified breadth-first search, *l* − *s*_*k*_ randomly selected nodes are added to the resulting induced subgraphs. The list of the induced subgraphs for the N81 and N90 networks are stored in the form of CSV files in which each row contains the node IDs included in the subgraph. Isolated nodes from the networks are not taken into account, and modules which are smaller than the predefined limit *l* are not included as induced subgraphs. Figure 2 presents a visualization for the truncation and search results.

All files created during preprocessing are inserted into pre-defined subfolders, along with a JavaScript Object Notation (JSON) file which contains the necessary input parameters for the further scripts.

### Machine learning model

**Fig. 3:**
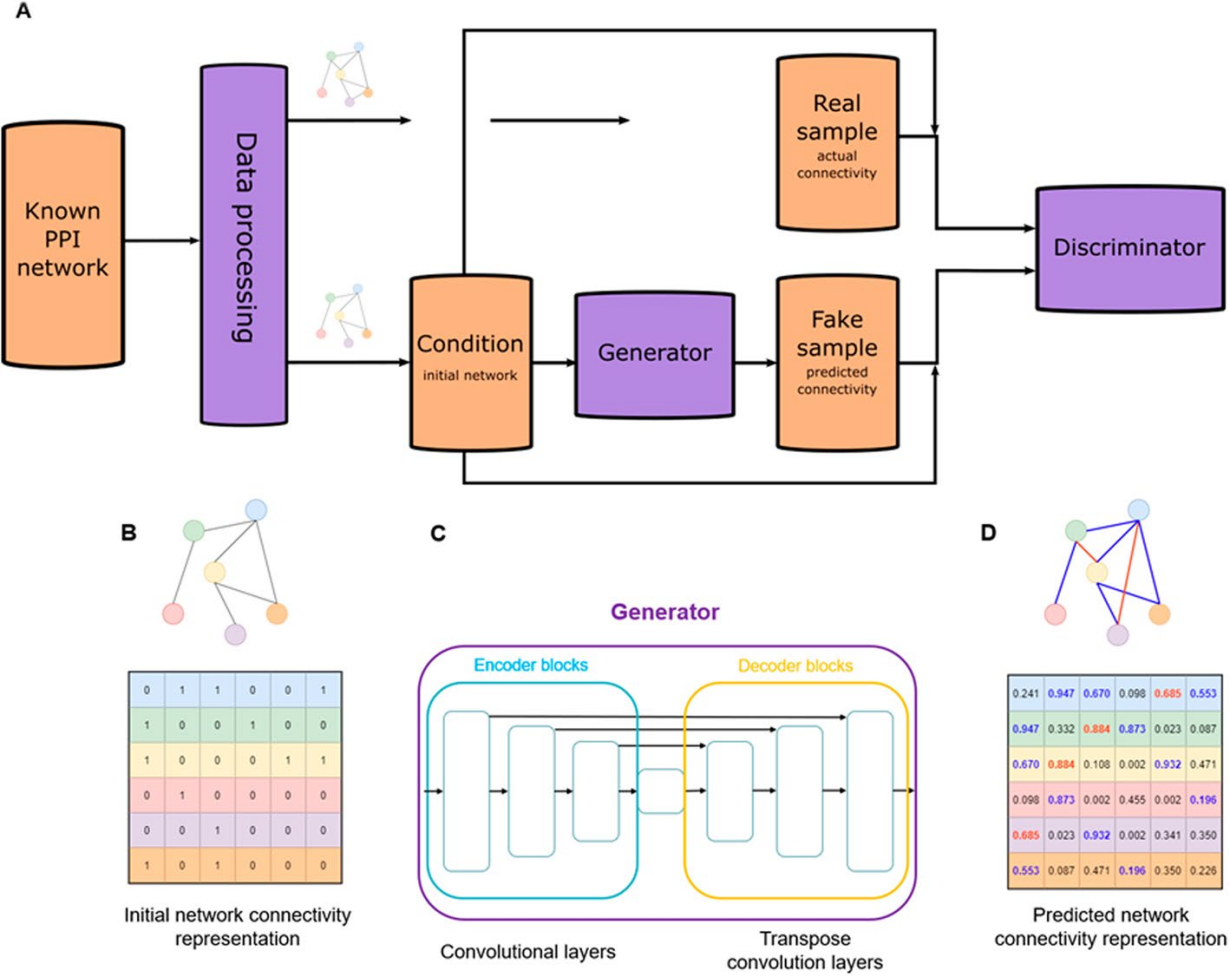
**Machine learning.** Schematic diagram of the conditional generative adversarial network (cGAN) architecture that uses the representation of the initial protein–protein interaction (PPI) network connectivity as condition with no input noise in the generator, and pairs of condition and real or generated connectivity representations in the discriminator (**A**) and simplified visualization of the prediction process (**B**–**D**). Input (sample condition) of the generator model is a representation of the initial connectivity via the adjacency matrix of the induced subgraph (**B**). Generator was implemented as a layer skipping concatenation method among the convolutional (encoder blocks) and transpose convolution (decoder blocks) layers (**C**). Output (fake sample) of the generator model is a representation of the predicted connectivity in the form of a confidence matrix, approximating the expected adjacencies in the given induced subgraph (**D**)

The second module of the link prediction tool trains a complex machine learning model, using the cGAN architecture as its basis (Fig. 3A). The model takes as input the initial representation of the given network, consisting of the adjacency matrices of the induced subgraphs from the lower connectivity level, i.e. the N81 network (Fig. 3A). During training, the generator aims to learn the mapping of the initial representation into the target representation, that consists of the adjacency matrices of the same node group from the higher connectivity level, i.e. the N90 network (Fig. 3C). For our generator, an encoder-decoder style network was implemented, based on the pix2pix GAN, where convolutional layers first encode the input into increasingly smaller feature maps, then transpose convolution layers decode them into increasingly larger ones [35]. The important novelty of the pix2pix architecture comes from the layer skipping concatenation of the encoded and decoded feature maps that carry over intact information from the initial representation. Our generator follows the same approach (Fig. 3B). However, it is paired with a convolutional network with Wasserstein distance-based loss [38] with gradient penalty [39] in the discriminator, instead of the PatchGAN based classifier used in the pix2pix work. Both networks in our cGAN model use the Adam optimizer (learning rate = 0.0002, beta_1 = 0.9, beta_2 = 0.99) and are trained for 3 epochs, in which for each training step the discriminator is allowed to execute its loss calculation and parameter optimization 5 times, then the generator executes the same for itself once per iteration. Parallelization is also introduced to both networks in the form of batch learning, with batch sizes of 64.

The model was implemented using Python version 3.7.10 with GPU enabled Tensorflow version 2.4.1 [40] and its inbuilt Keras packages [41]. During trainings, the provided network data are first processed by extracting the corresponding adjacency matrices for each induced subgraph and building the initial network representations of both the N81 and N90 networks. These are paired with the adjacency matrices of the N90 and N100 networks, respectively, as their target representation. Training proceeds by using the initial representation of the N81 network as the condition and the N90 target representation as the real sample for the cGAN. The data of the N90-N100 pair is used for performance tracking purposes at given intervals. For the data preprocessing in Python, process-based parallelism was implemented using the multiprocessing library, while GPU parallelism might be utilized, given a CUDA compatible NVIDIA card for the training.

### Using the trained model

As described previously, there are two different ways of using our software, depending on the intended use of the results. One serves as the evaluation tool (which can be found in gan_90.py in our GitHub repository), producing results for comparison with the original network (N100) providing performance measurement metrics. The model primarily uses the N81 network and learns to approximate the adjacencies of the N90 network. After the training is complete, the model is called to use the N90 network representation and provide predictions for the N100 network, writing the link confidences into an output file, that is later processed via selecting the maximum value for the repeating node pairs. Predictions for the already existing links are pruned, making evaluation measurements more trivial to run on these outputs.

The other option (provided in gan_100.py) learns directly on the N90 network representation to approximate the N100 adjacencies. This model is intended to be used for predicting entirely unknown edges (~ N110) which are evaluated via experimental methods, rather than using a more complete database. The output of this model is generated in the same structure as for the previous one.

All scripts for the link prediction software along with the detailed parameters used in network training are available at https://github.com/semmelweis-pharmacology/ppi_pred.

## Results

We developed a software that takes the adjacency list of an arbitrary network, processes the topology via a modified breadth-first search to provide smaller induced subgraphs, and trains a cGAN model conditioned on these inputs. The resulting generator neural network is capable of performing link prediction on a higher connectivity level for the same network it used during training, for which it provides a list of aggregated confidence values as the probabilities of each node pairs forming a new link. To evaluate the performance of our prediction tool, we used datasets containing physical protein–protein interactions of *Homo sapiens*, *Saccharomyces cerevisiae* (yeast), *Mus musculus* (mouse), *Rattus norvegicus* (rat), and *Sus scrofa* (boar) from the STRING (Search Tool for the Retrieval of Interacting Genes/Proteins) database. STRING is a publicly available web resource, containing information on protein–protein interactions, both physical and functional [42], from which our evaluation set was extracted and filtered based on the combined STRING-score with a cutoff confidence value of ≥ 0.95. The scores, summarized in Table 1, were generated by taking the mean of the results of tenfold cross-validations for each network. These tests show promising performance with consistently high values and a low variance across the different species, which suggests good generalization of our model, that is further supported by the comparison of our method to relevant approaches as described in the discussion section. Training and test runs were performed on an ASUS RS720-E9-RS8-G server (CPU: 2 × Intel Xeon Gold 5218, 16-core, 2.30 GHz; RAM: 512 GB; Tesla V100 PCIe 32 GB) with Ubuntu Server 20.04.1 LTS operating system.

**Table 1:** Mean results across tenfold cross-validation of prediction for each investigated species

Species	AUROC	AUPRC	NDCG	Computing time (s)
*Homo sapiens*	0.913	0.169	0.761	1310
*Saccharomyces cerevisiae*	0.931	0.202	0.781	899
*Mus musculus*	0.909	0.137	0.742	1334
*Rattus norvegicus*	0.925	0.252	0.809	1525
*Sus scrofa*	0.898	0.120	0.721	1429
Mean	0.915	0.176	0.763	1299

AUROC: area under the receiver operating characteristic curve; AUPRC: area under the precision-recall curve; NDCG: normalized discounted cumulative gain

In order to demonstrate the robustness of our original cGAN-based model (titled “Adjacency only” version), we also prepared two alternative implementations of our prediction tool. In the first (titled “Embedding only” version), we replaced the adjacency matrices of the induced subgraphs in the input (initial network representation) with node embedding vectors of the corresponding nodes, that created same-size input matrices. We used the natural language processing inspired node2vec algorithm [43] (Additional file 1: Fig. S1) to generate embedding vectors of all nodes on the different network connectivity levels. In the second alternative implementation (titled “Combined” version), we combined the adjacency matrices of the induces subgraphs with the embedding vectors of the corresponding nodes via simply concatenating the resulting two matrices. Comparison of results for the 3 different approaches on the same 3 metrics (AUROC, AUPRC, NDCG) are summarized in Table 2 and pairwise comparisons by Mann–Whitney–Wilcoxon tests on them are presented in Table 3. The scores show that the embedding only inputs performed significantly worse, than both other methods, while the combined approach resulted in no significant performance change (Tables 2, 3), see the Additional file 1: Fig. S2 for detailed boxplots. Thus, the inner representation of topological features of the cGAN is robust enough to depend only on raw adjacency-based input data, and does not rely on additional node embedding algorithms.

**Table 2:** Mean results across tenfold cross-validation of prediction for each investigated species with the 3 different input data type: adjacency matrices only, adjacency matrices concatenated with embedding vector-based matrices, embedding vector-based matrices only

Species	Adjacency only			Combined			Embedding only		
	AUROC	AUPRC	NDCG	AUROC	AUPRC	NDCG	AUROC	AUPRC	NDCG
*Homo sapiens*	0.913	0.169	0.761	0.915	0.179	0.767	0.761	0.022	0.606
*Saccharomyces cerevisiae*	0.931	0.202	0.781	0.930	0.210	0.787	0.789	0.034	0.635
*Mus musculus*	0.909	0.137	0.742	0.904	0.135	0.739	0.739	0.020	0.599
*Rattus norvegicus*	0.925	0.252	0.809	0.923	0.258	0.812	0.750	0.027	0.633
*Sus scrofa*	0.898	0.120	0.721	0.895	0.126	0.729	0.745	0.017	0.583
Mean	0.915	0.176	0.763	0.913	0.182	0.767	0.757	0.024	0.612

AUROC: area under the receiver operating characteristic curve; AUPRC: area under the precision-recall curve; NDCG: normalized discounted cumulative gain; Combined: adjacency matrices concatenated with embedding vector-based matrices as input

**Table 3:** Comparisons of the results from Table 2, presenting q-values from Mann–Whitney–Wilcoxon tests

Species	Adjacency only–combined			Adjacency only–embedding only		
	AUROC	AUPRC	NDCG	AUROC	AUPRC	NDCG
*Homo sapiens*	0.297	0.577	0.579	< 0.001	< 0.001	< 0.001
*Saccharomyces cerevisiae*	0.971	0.631	0.631	< 0.001	< 0.001	< 0.001
*Mus musculus*	0.523	0.796	0.688	< 0.001	< 0.001	< 0.001
*Rattus norvegicus*	0.228	0.481	0.475	< 0.001	< 0.001	< 0.001
*Sus scrofa*	0.429	0.429	0.290	< 0.001	< 0.001	< 0.001
Mean	0.490	0.583	0.533	< 0.001	< 0.001	< 0.001

AUROC: area under the receiver operating characteristic curve; AUPRC: area under the precision-recall curve; NDCG: normalized discounted cumulative gain; Combined: adjacency matrices concatenated with embedding vector-based matrices as input

Due to the use of induced subgraphs and downscaling in the networks, nodes from isolated network components with size below the subgraph node limit (*l* = 36) were left out of the preprocessing, and consequently their links cannot be predicted by the model. To evaluate the possible limiting effects of this phenomenon in practice, we summarized the properties of the PPI networks along with the ratio of node coverage of the induced subgraph generating algorithm and the ratio of expected links that failed to receive any confidence values during prediction (Table 4).

**Table 4:** Basic network properties and the limiting effects of using induced subgraphs for novel link prediction, for each investigated species

Species	Number of proteins (nodes)	Number of interactions (links)	Network sparsity with self-loops included (%)	Induced subgraph node coverage on N90 (%)	Induced subgraph node coverage on N100 (%)	Known missing links that could not be predicted in N100 from N90 (%)
*Homo sapiens*	3555	16,586	0.26	67.2	69.8	12.3
*Saccharomyces cerevisiae*	2052	16,635	0.79	77.8	79.3	6.5
*Mus musculus*	3868	16,548	0.22	64.0	66.5	14.7
*Rattus norvegicus*	4053	22,249	0.27	68.5	71.2	10.1
*Sus scrofa*	3731	14,637	0.21	68.9	71.5	15.3
Mean	3452	17,331	0.28	69.3	71.7	11.8

To demonstrate the utility of our cGAN model-based PPI prediction tool in comparison with node embedding-based pairwise classifiers, we tested the node2vec and struc2vec utilizing methods of Yue et al. [27] with the same PPI networks that were used for our results. Even with an 80/20% network downscaling ratio (instead of 90/10%), the cGAN model consistently outperformed both the node2vec and struc2vec versions (AUROC: 0.907 vs 0.819 and 0.864, respectively). See Additional file 1: Table S2 and its supplementary method description in Additional file 1 for additional details. To further investigate these results, we performed Gene Ontology (GO) enrichment analysis with semantic similarity measurements on the predicted PPIs from the node2vec and struc2vec classifiers of Yue et al. [27] and our PPIs from the model with 80/20% downscaling ratio. The analysis revealed, that despite the enriched GO term sets derived from the 3 different methods are partly similar, several GO terms are occurring exclusively in our results (Additional file 1: Fig. S3). Although our approach is based on topological information only, biological evidence may suggest that the false positive results in our predictions correspond to yet unknown edges in the interactomes. In order to validate the false positive edges we predicted, we also performed GO enrichment analysis [44, 45] on our original predictions (Table 2), along with gene and transcript length analysis thus mapping the characteristics of true positive and false positive edges. Enriched GO terms from all species, separately for true positive and false positive sets, and ontology categories were combined into Venn diagrams to highlight similarities and differences between true positive and false positive sets (Additional file 1: Fig. S4, Table S3 and S4). The results show characteristic patterns in form of common enriched GO terms occurring in constant manner cross-species in both true positive and false positive sets in all 3 investigated ontologies. The length analysis showed false positive protein coding genes and transcripts to be constantly longer in case of all examined species (Additional file 1: Table S5).

**Fig. 4:**
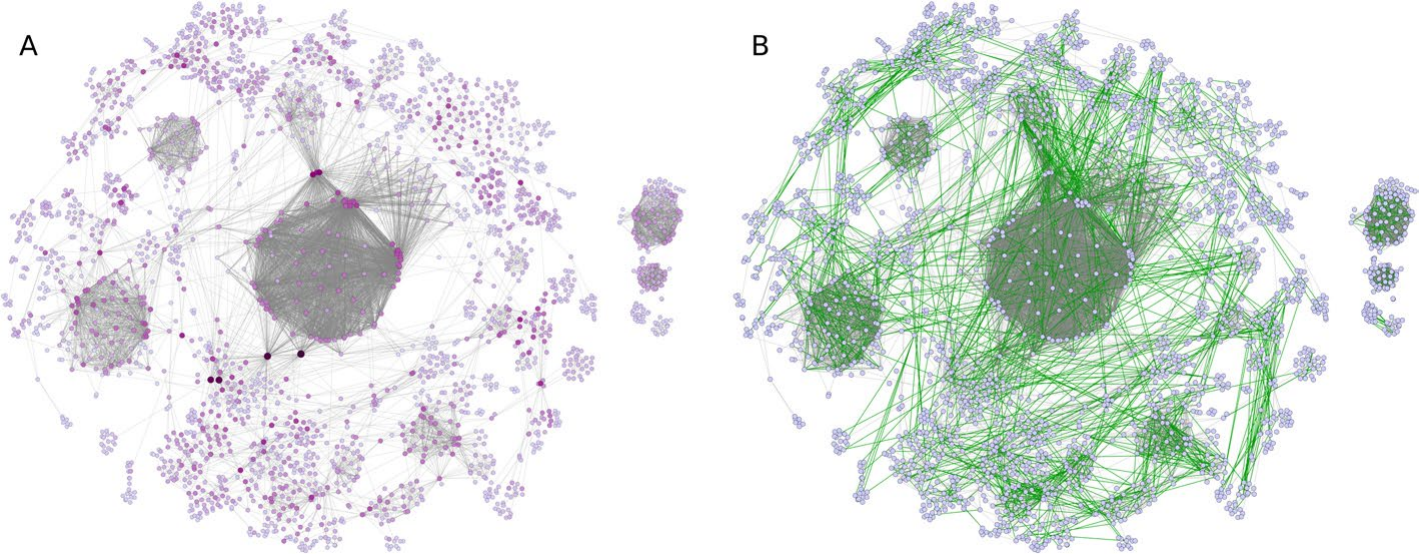
**Visualizations of results: modified breadth-first search (BFS) coverage and predictions.** Coverage of network representing an example truncated human protein–protein interaction (PPI) training dataset (N90) achieved by modified BFS. Node size and color intensity represent the frequency of occurrence of the given node in induced subgraphs assessed by the modified BFS (**A**). Human PPI network with green edges denoting the top predicted interactions to complete the truncated N90 network to an N100 network (**B**). Only network components covered by modified BFS are shown in these representations (giant component and appropriately sized isolated components). See Additional file 2: Fig. S5 for high-resolution image of the representative initial full network with node annotation

To better illustrate the prediction process of our model, Fig. 4 presents the visualization of the coverage (and overlapping) of the induced subgraphs (Fig. 4A) and the top expected number of predicted links with the highest confidence values (Fig. 4B) in one of the tenfold test runs for the human PPI network. See also Additional file 2: Fig. S5 for a high-resolution image of the representative initial full network with node annotation. All network visualizations were generated by the Cytoscape network analysis framework with the use of the EntOptLayout plugin after preordering by the Prefuse force-directed layout algorithm [46, 47].

All PPI networks used in this study are uploaded to our GitHub repository, in line with the license of the original database. Additionally, all predicted PPIs by our cGAN model are provided in Additional files 3–7: Datasets S1–S5 (results for *Homo sapiens*, *Saccharomyces cerevisiae*, *Mus musculus*, *Rattus norvegicus*, and *Sus scrofa*, respectively), however further validation should be undertaken before their practical use.

## Discussion

Here, we presented a software that implements a complex generative machine learning framework based on the cGAN architecture, that trains an artificial neural network, called the generator, to perform link prediction in PPI networks.

Machine learning models can be categorized into generative and discriminative models based on their approach of modelling the provided data. A discriminative model (like logistic regression, SVM, neural net classifier) learns specifically to distinguish the underlying data distribution of the given classes, meanwhile a generative model (like auto-encoders, GANs, neural language models) learns the structure of the entire data distribution directly via generating similar data. Discriminative models are more intuitive and work exceptionally well with quality features, however, they require a large set of labeled samples to accurately represent the real world.

The PPI prediction task is traditionally approached via various pairwise classification methods [10–17] that fall into the discriminative model category, which require supervised training, meaning that positively as well as negatively labeled training data have to be provided in order to optimize the parameters of the model and to generalize the separation of the two differing distributions to real world unseen data. The choice of classifier determines the required size of the training data, while also limiting the capacity of the model to properly discover the feature space. Artificial neural networks serve as the basis of several well-performing and widely used classifiers [14–17], providing high information capacity and inherent feature processing capabilities, that ease the difficulties of finding the best information representations. Consequently, neural network-based classifiers perform best with large-scale datasets (i.e. having millions of samples), that accurately represent the world as its subsample. In the case of network based PPI data, the known interactome is greatly sparse (0.2–0.8% of all possible links are present in the STRING networks [42]), making the available positive data arguably insufficient, while the negative data (negatome [48]) is barely available in relevant quantities. Due to the lack of confirmed negative data, negative sampling is used to randomly (or via heuristics) select samples from the unlabeled data and label them as negative, until the number of validated positive and randomly selected negative samples create a balanced dataset. Then, this set is divided to a training and a testing split, or into multiple splits using the k-fold method to address variance in the data. The performance of the trained classifier is determined by its scores on the withheld test set, that carries the limitation of negative sampling the same way as the training set does. Thus, the score of the classifier might get hindered [49], as it mostly learns the distinction between the positively labeled and the entirely unlabeled data samples or alternatively, it models the heuristic method used to create the negative labels.

We can observe, however, the widespread use of random negative sampling methods even in recently published discriminative model-based PPI prediction approaches [27, 50], that introduce an arguable theoretical shortcoming in the evaluation of their true performances as well as doubts about their applicability outside of their experimental domain. Generative machine learning models aim to mitigate the lack of labeled data via letting the model discover the classes in the underlying distribution by itself. Here, we provided a generative machine learning model, that is free from the worries of random negative sampling. Yue et al. [27] utilized 12 different network embedding algorithms for node feature generation, and evaluated their applicability for PPI prediction with a simple logistic regression model, on the human PPI network from the STRING database (cutoff confidence 0.7). In order to provide the best possible basis of comparison, we changed the downscaling ratio in our model to 80/20% (instead of 90/10%) to better match the 80/20% training–testing set split in their work. Then, we ran their implementation of node2vec and struc2vec utilizing pairwise classifiers with the same PPI networks (STRING cutoff 0.95) that we used. Comparison with these results shows that our purely adjacency-based generative approach consistently outperforms network embedding-based classification methods that use random negative sampling (AUROC = 0.819 and 0.864 by Yue et al. [27] node2vec and struc2vec respectively vs. AUROC = 0.915 by our model). See Additional file 1: Table S2 for further details. Our PPI prediction approach can also be compared to the pairwise classifiers that do not operate on network-based features, but rather they rely on protein attributes from external databases, like physicochemical properties, domain knowledge, gene ontology annotations, or even the protein sequences themselves. The use of these laborious quality features could severely limit the availability of training data and consequently the generalization capability and scalability of the model outside of the experimental domain. For example, Hashemifar et al. utilized the probabilistic evolutionary profile matrix of the proteins, which was generated by using multiple sequence alignments and web-based protein database search, in order to provide input features for a Siamese-like convolutional neural network classifier [50]. They applied random negative sampling as well, and reported tenfold cross-validation on benchmark human (obtained from Hippie v1.2) and yeast (derived from DIP) PPI data, that resulted in AUPRC values of 0.413 and 0.467 respectively. Our method, which is strictly topology-based and consequently independent of external databases, was evaluated on the entire human and yeast PPI network data, with large label imbalances (link sparsity) in the testing set, and still managed to score AUPRC values of 0.169 and 0.202 respectively, showing the potentials of our generative machine learning model.

Generative models, however, are not without their own limitations, as in order to optimize the generation of quality data, the model requires a loss function that properly measures the differences from the ground truth. Such a loss function is difficult to determine, requiring handcrafted functions by experts, as traditional functions, like Euclidian distance, often fail to satisfy human quality evaluation. The novelty of GANs lies in the way they overcame this obstacle. The GAN architecture includes two neural networks: the generator, which is the main unsupervised generative model, and the discriminator, which provides the objective for the generator, creating a supervised environment for the training of an otherwise unsupervised model. Consequently, the generator automatically learns a loss function appropriate for satisfying a higher-level goal, which in this case is to approximate the given data distribution via the generated samples. In our work, the task of the generator is to generate proper adjacency matrices on higher connectivity level, given a prior distribution as a conditional guidance (like the outlines for an image to be filled in) in the form of the incomplete adjacency matrices of the same region in the network with lower connectivity. In order to optimize this generation process, the discriminator model is given the task of properly classifying the prior and generated adjacency matrix pairs as fake, while also classifying the provided prior and actual complete adjacencies as real. The loss of the generator is tied to the classification error of the discriminator, which the generator tries to maximize. The loss of the discriminator is also this same error, which the discriminator tries to minimize. During their mini-max game, both networks improve in their tasks, but it is important to note that after the training is finished, only the generator network is used in practice.

GANs are recent and popular subjects among unsupervised learning models, and especially among generative models, however, our choice of prediction approach was further motivated by the performance of GANs in both image-to-image translation task and the prediction of network evolution in dynamic social networks [32, 33]. The implemented cGAN explores the behavior of connectivity forming inside the provided network through simulating a temporal evolution, and generalizing the knowledge to further expand its links. The method can be expanded in both ends, with more levels of downscaling to provide a more elaborate dataset of initial and target snapshots, as well as to rerun the whole model on the more complete network repeatedly to gradually discover more and more links. The use of random uniform downscaling ensures that we introduced as little bias into the simulated temporal evolution as possible. Using the real evolution of the PPI networks instead would mean that a GAN model might only learn the manner in which researchers validated the PPIs throughout the years, and so would lead to an arguable bias.

An important limitation of the original GAN-based networks was that they were infamously unstable and had difficulties reliably converging to an optimal state [28, 38, 39]. To reach the optimal state during training, either the discriminator or the generator should stay slightly ahead of its counterpart in regards of performance, so that the latter is able to learn from the advances of the former via a non-zero gradient of the loss function. Their pace of becoming increasingly more complex should be of similar rate, letting neither of them defeat the other, as that might halt the training process permanently. In case of the generator running out of balance, a phenomenon called mode collapse will cause the model to be unable to learn further significant details, while in case of the discriminator learning too quickly, the vanishing gradients will result in the same problem. To counter these extreme outcomes, the loss function of the discriminator is commonly switched out to a Wasserstein distance-based approach. In the Wasserstein GAN (WGAN) model, the discriminator operates as a “critic”, scoring the input samples according to their measure of realness, creating a continuous and differentiable cost for the training that produces clean, non-zero gradients [38, 39]. Such optimization algorithm, in fact, appropriately approximates the maximization task of the Wasserstein distance between real and fake samples in respect to the discriminator, as well as the minimization task of the same distance in respect to the generator. However, it must be noted that the approximation requires the discriminator to operate as a 1-Lipschitz function, for which certain constraints have to be enforced. In the original WGAN model, weight clipping was utilized to achieve Lipschitz continuity [38], that was later improved upon by other methods, like gradient penalty and spectral normalization. For our discriminator network, we implemented gradient penalty with a penalty coefficient *λ* = 10, as recommended by its authors [39].

The modifications made to the discriminator represent only but a small fraction of the neural net architectures used in GAN models, for which both participating networks have a seemingly endless, already established alternatives in literature [28–35]. Our approach relied on the highly specialized generator model from the pix2pix GAN [35], which is an encoder-decoder styled network, utilizing deep convolutional layers that are well-fitted for image processing tasks, but may fall behind with other forms of data representation. As a result, further improvements may lie in the investigation of network alternatives for both the discriminator and the generator, as well as the way they are interacting during training and the input data is presented to them.

The GO enrichment analysis comparing the results of our cGAN-based model with those of other prediction methods (Yue et al. [27] node2vec and struc2vec) suggests, that by having GO terms that are uniquely enriched in cGAN result set, the predicted new edges might have biological relevance, thus highlighting the utility of using cGAN for PPI prediction (Additional file 1: Fig. S3). GO enrichment analysis results comparing true positive and false positive edges allow us to hypothesize that if proteins (nodes) connected by the predicted false positive edges bears characteristics similar to the true positive ones, those false positive edges might denote a not yet known but indeed existing interaction (Additional file 1: Fig. S4, and Additional file 1: Table S3 and S4). The gene and transcript length analysis results indirectly bears information on evolutionary conservation thus suggests that our setup might tend to predict edges connecting evolutionarily more conserved protein coding genes [51] (Additional file 1: Table S5).

Properly describing the information of a network in a machine learning-compatible representation is not a trivial task. Complete adjacency matrices of large-scale networks could easily result in matrix dimensions of tens of thousands, rendering the data unusable for a single input due to technical limitations, like insufficient GPU memory capacity, as well as computational limitations, like difficulty for a similarly scaled model to converge. Thus, the network has to be either reduced in representation or divided into smaller parts, that are created in similar fashion and possess similar information about the network as a whole. The breadth-first search algorithm [37], given a node limit, can explore the neighborhood of the starter node and provide an induced subgraph of the traversed region, generating local adjacency matrices for each starting node. However, the limitation of the algorithm should be noted, that in order to satisfy the node limit, the algorithm has to stop often in the middle of a given depth layer. We aimed to avoid any sort of bias in the selection of nodes in the last layer by randomly assigning as many of them to the search results as the limit allowed. Furthermore, the generation of induced subgraphs results in the obvious trade-off problem of eliminating isolated nodes or network components, that are below the required number of connected nodes (limit *l* = 36). Consequently, no links can be predicted between nodes that are not present in the same induced subgraph of the initial level of connectivity, i.e. they have either no valid path connecting them or the shortest path is 37 or more units of distance. The performance limiting effect of the induced subgraph generation can be characterized with the percentage of node coverage on the N90 and N100 networks and the ratio of “unpredictable” links that were expected to be predicted from N90 to N100, but were missing a confidence score in the results, because the model did not encounter an induced subgraph with them. Despite of the node coverage of the algorithm being relatively low (64–78% and 66–79%, see Table 4), the ratio of unpredictable links across the species (7–15%, see Table 4) indicates that the low node coverage of N90 is not detrimental to the performance of the method. Moreover, the higher average node coverage of the N100 networks might also mean an even lower ratio of non-predictable, yet highly probable links in practice, when the aim is to predict novel interactions from the original N100 network. However, to properly analyze this, we would need a large scale experimental evaluation of the results.

## Conclusion

We developed a software that implements a link prediction tool for the purpose of PPI prediction utilizing machine learning. Molecular attributes from external databases are not required for our software, as only raw topological features are used in the conditioning of a generative adversarial machine learning model, that learns to predict new probable links in the protein–protein interaction network. The evaluation of our software serves as the first demonstration of the applicability of a cGAN model for the PPI prediction task, as well as its structure could provide a basic framework for future link predictor tools that involve generative models.

## Availability and requirements

Project name: ppi_pred

Project home page: https://github.com/semmelweis-pharmacology/ppi_pred

Operating system(s): GNU/Linux

Programming language: Python, Bash shell scripts

Other requirements: Python 3.7.10, Tensorflow 2.4.1 (or the GPU-enabled version with Cudatoolkit 10.1.243), Pandas 1.2.4, Numpy 1.20.2

License: MIT

Any restrictions to use by non-academics: N/A

## Supplementary Information

**Additional file 1**: **Supplementary Materials.** Includes Figures S1–S4., Tables S1–S5., and the corresponding method descriptions.

**Additional file 2**:** Figure S5. **STRING database derived annotated human protein-protein interaction network. High-resolution human protein–protein interaction network from the STRING database (cutoff 0.95) with annotation of nodes with protein coding gene symbols and visualization of isolated components which could not be included in the analysis due to limitations.

**Additional file 3: Dataset S1**. Prediction results for *Homo sapiens*. Unvalidated human PPI predictions of the cGAN model, which was trained to predict the N100 connectivity from the N90 level, then was used to produce new predictions (~ N110) from the N100 level. See the Implementation section for more details. STRING protein identifiers are mapped to preferred gene names according to information in the STRING database.

**Additional file 4: Dataset S2**. Prediction results for *Saccharomyces cerevisiae*. Unvalidated yeast PPI predictions of the cGAN model, which was trained to predict the N100 connectivity from the N90 level, then was used to produce new predictions (~ N110) from the N100 level.33See the Implementation section for more details. STRING protein identifiers are mapped to preferred gene names according to information in the STRING database.

**Additional file 5: Dataset S3**. Prediction results for *Mus musculus*. Unvalidated mouse PPI predictions of the cGAN model, which was trained to predict the N100 connectivity from the N90 level, then was used to produce new predictions (~ N110) from the N100 level. See the Implementation section for more details. STRING protein identifiers are mapped to preferred gene names according to information in the STRING database.

**Additional file 6: Dataset S4**. Prediction results for *Rattus norvegicus*. Unvalidated rat PPI predictions of the cGAN model, which was trained to predict the N100 connectivity from the N90 level, then was used to produce new predictions (~ N110) from the N100 level. See the Implementation section for more details. STRING protein identifiers are mapped to preferred gene names according to information in the STRING database.

**Additional file 7: Dataset S5**. Prediction results for *Sus scrofa*. Unvalidated pig PPI predictions of the cGAN model, which was trained to predict the N100 connectivity from the N90 level, then was used to produce new predictions (~ N110) from the N100 level. See the Implementation section for more details. STRING protein identifiers are mapped to preferred gene names according to information in the STRING database.

## Data Availability

The source code of the presented software is available at https://github.com/semmelweis-pharmacology/ppi_pred. Further details about the test results and the methodology are available from the authors on reasonable request.

## Abbreviations

AUPRC: Area under the precision-recall curve
AUROC: Area under the receiver operating characteristic curve
cGAN: Conditional generative adversarial network
CSV: Custom separated value
GAN: Generative adversarial network
JSON: JavaScript object notation
NDCG: Normalized discounted cumulative gain
PPI: Protein–protein interaction
STRING: Search tool for the retrieval of interacting genes/proteins
SVM: Support vector machine
WGAN: Wasserstein generative adversarial network
