# Supplementary material for: Efficient link prediction in the protein–protein interaction network using topological information in a generative adversarial network machine learning model
Source: BMC Bioinformatics. 2022 Feb 19;23:78. doi: 10.1186/s12859-022-04598-x (PMC8858570; doi:10.1186/s12859-022-04598-x)
Supplement: Supplementary file 1 — Additional file 1: Supplementary Materials. Includes Figures S1–S4., Tables S1–S5., and the corresponding method descriptions. [file 12859_2022_4598_MOESM1_ESM.pdf]

Efficient link prediction in the protein-protein interaction network using topological information in a generative adversarial network machine learning model

Supplementary materials

Olivér M. Balogh[1,2], Bettina Benczik[1,3], András Horváth[2], Mátyás Pétervári[1], Péter Csermely[4], Péter Ferdinandy[1,3], Bence Ágg[1,3]

[1] Cardiometabolic and MTA-SE System Pharmacology Research Group, Department of Pharmacology and Pharmacotherapy, Semmelweis University, Budapest, Hungary

[2] Faculty of Information Technology and Bionics, Pázmány Péter Catholic University, Budapest, Hungary

[3] Pharmahungary Group, Szeged, Hungary

[4] Department of Molecular Biology, Semmelweis University, Budapest, Hungary

Corresponding author: Bence Ágg, MD, PhD
Email: agg.bence@med.semmelweis-univ.hu
Phone: +36 30/634-23-49
Address: Nagyvárad tér 4., 1089, Budapest, Hungary

Table of contents

1. Using embedding features for alternative implementations 3
2. Comparison of cGAN with embedding-based pairwise classifiers... 6
3. Gene ontology analysis of the predicted protein-protein interactions 7
4. Gene and transcript length analysis... 12
References 14

1. Using embedding features for alternative implementations

Instead of the original model ("Adjacency only"), we investigated two alternative models: one which utilizes embedding features only ("Embedding only"), and one which concatenates the adjacency matrices with these corresponding embedding vectors ("Combined"). In both cases, the embedding is executed in the preprocessing module (Supplementary Fig. 1). We chose the popular node2vec algorithm, which is a natural language processing inspired embedding method, that reduces the representation space of nodes by learning low-dimensional features based on their neighborhoods in the graph [1]. We used the out-of-the-box, high performance C++ implementation of the node2vec algorithm, that is included in the libraries of the Stanford Network Analysis Platform (SNAP) [2]. Parametrization of the learning followed the values presented in Supplementary Table 1, with a carefully selected number of embedding dimensions in the output representation, which was set to match the number of nodes in the induced subgraphs in order to construct matching square matrices that can be concatenated later on in the main machine learning part. Statistical analysis of results for the different model types are summarized *via* boxplot representation in Supplementary Fig. 2. Kruskal-Wallis test was used to compare performance of the investigated two alternative models and the original model. Post hoc comparisons were performed using the Mann-Whitney-Wilcoxon test. For both tests significance level was set to 0.05 and false discovery rate (FDR) correction was used to adjust p-values for multiple testing.

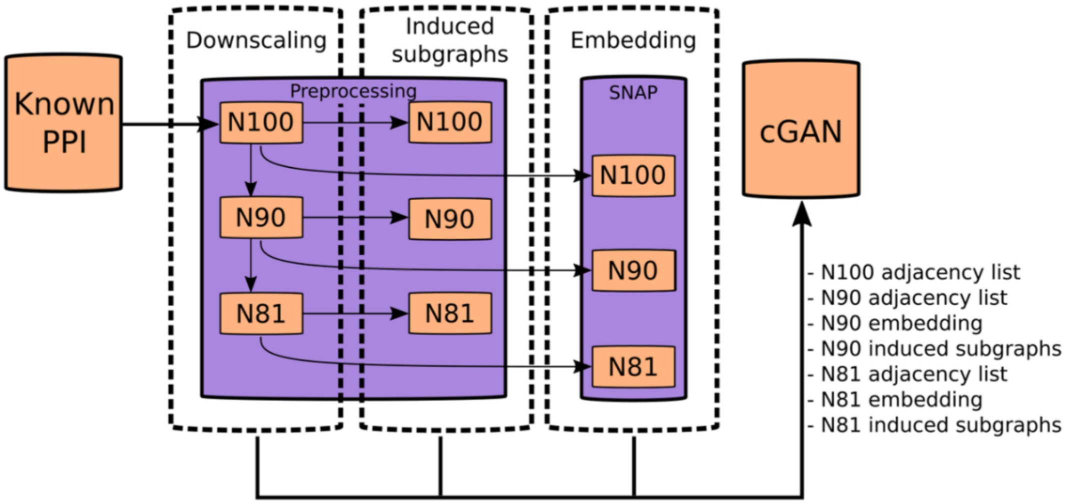

Supplementary Figure 1: Preprocessing of the input network for the combined model. Schematic summary of the preprocessing module in the combined model, that takes in the provided protein-protein interaction (PPI) network, and produces the downscaled networks with 90% (N90) and its 90% (thus 81%, N81) of edges from the original one (N100) in the form of adjacency lists, and generates the induced subgraphs as well as the node embeddings for each. These representation files are created for the original network as well but are not required in the machine learning part, resulting in the listed 7 files to be fed into the conditional generative adversarial network (cGAN) model down the line.

Supplementary Table 1: The parametrization of the node2vec algorithm.

Number of dimensions (d)	32
Length of the walk per source (l)	80
Number of walks per source (r)	10
Context size for optimization (k)	10
Number of epochs in SGD (e)	5
Return hyperparameter (p)	0.25
In-out hyperparameter (q)	0.25

Parameter abbreviations used in the code are in brackets, ***SGD****: stochastic gradient descent*

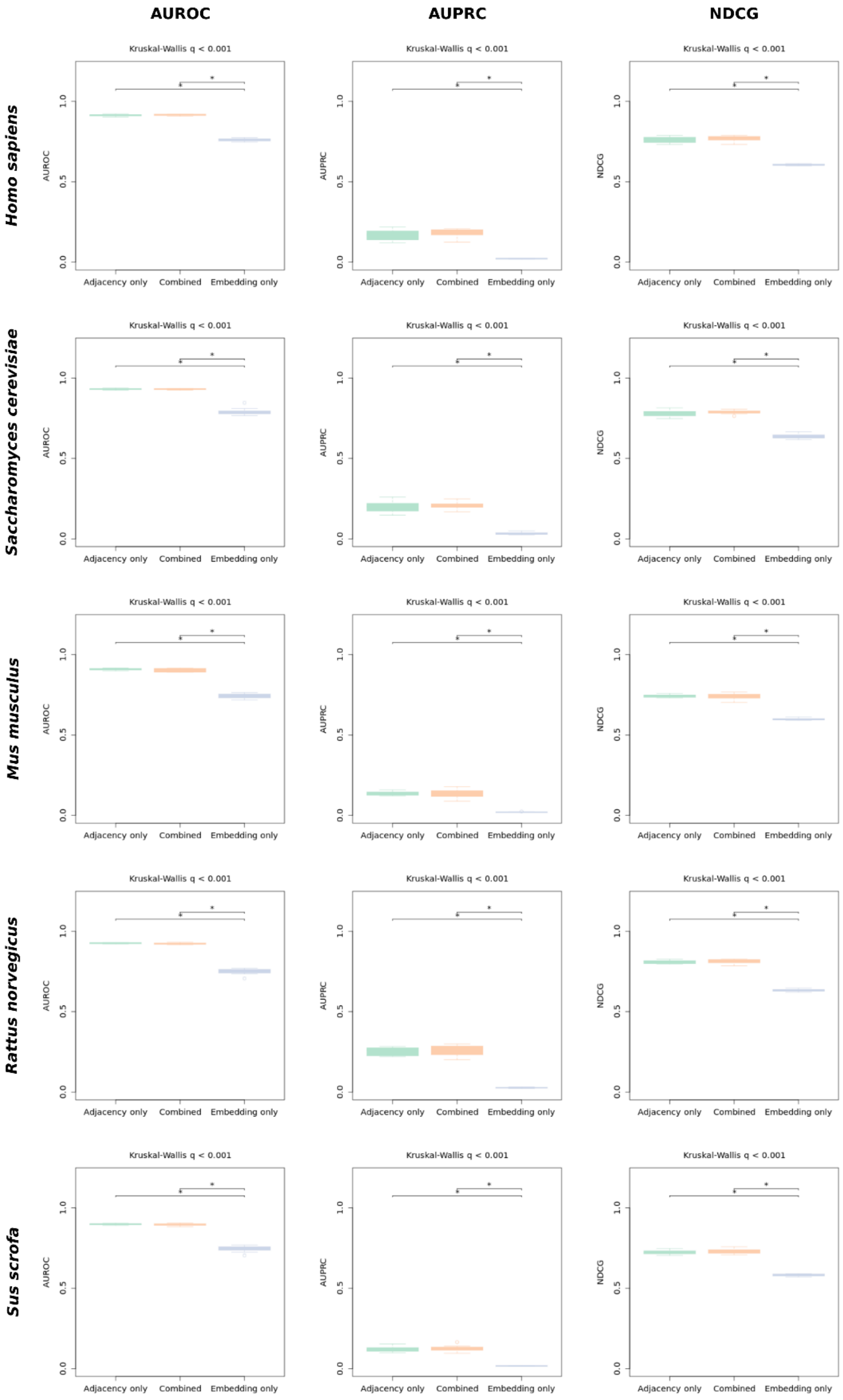

Supplementary Figure 2: Comparison of alternative implementations of our prediction tool with the original cGAN-based model. **AUROC**: area under the receiver operating characteristic curve, **AUPRC**: area under the precision-recall curve, **NDCG**: normalized discounted cumulative gain, **Combined**: adjacency matrices concatenated with embedding vector-based matrices as input, ***:** q-value based significance

2. Comparison of cGAN with embedding-based pairwise classifiers

Supplementary Table 2: Comparison of our results with node embedding based pairwise classifiers, via the implementation of Yue et al. [3]. We chose to use the same metrics which were presented in their work.

Species	cGAN 90/10% downscaling		cGAN 80/20% downscaling		Yue et al., node2vec		Yue et al., struc2vec	
	AUROC	Accuracy	AUROC	Accuracy	AUROC	Accuracy	AUROC	Accuracy
H. sapiens	0.913	0.931	0.906	0.872	0.827	0.755	0.869	0.805
S. cerevisiae	0.931	0.928	0.925	0.874	0.847	0.778	0.888	0.820
M. musculus	0.909	0.925	0.900	0.863	0.811	0.739	0.859	0.794
R. norvegicus	0.925	0.925	0.916	0.865	0.822	0.751	0.877	0.813
S. scrofa	0.898	0.927	0.889	0.875	0.788	0.719	0.829	0.768
Mean	0.915	0.927	0.907	0.870	0.819	0.748	0.864	0.800

***cGAN:** conditional generative adversarial network **AUROC**: area under the receiver operating characteristic curve*

In order to provide the closest comparison setup possible, we prepared our results with a different downscaling ratio to match the 80/20% training-test set ratio used by Yue et al. in their methods. Although the 80/20% downscaling ratio in our work does not correspond to the concept of train/test ratio in the cross validation of pairwise classifiers, we applied it anyway to better resemble the conditions of the referred methods. Consequently, the preprocessing steps in our prediction tool produced N80 and N64 networks, of which the prediction from N64 to N80 was trained to the conditional generative adversarial network (cGAN) model, and the prediction from N80 to N100 was tested across the species. We used our PPI networks (STRING cutoff 0.95 [4]) for both the cGAN and the methods of Yue et al., instead of the ones they used (STRING cutoff 0.7) in order to create the most accurate comparison possible. We applied a 5-fold cross validation setup for our cGAN (due to the 80/20% downscaling ratio), however, the implementation of Yue et al. did not allow us to evaluate the performance of their node2vec and struc2vec based classifiers in a k-fold manner, and thus the results of only a single prediction were used. Results, summarized in Supplementary Table 2, show a consistent improvement in performance for our model as compared to the node embedding based classifiers.

3. Gene ontology analysis of the predicted protein-protein interactions

Using the results summarized in Supplementary Table 2, we also performed an analysis of biological functionalities among the proteins of the predicted interactions from cGAN, node2vec and struc2vec. For each species node2vec and struc2vec predictions were filtered based on the confidence score with a cutoff value >0.5, while from our cGAN results, we selected the top 20% highest confidence scores produced by one prediction from N80 to N100, as that resembles the intended use of the model in practice. We generated the analysis inputs from filtered positive edges represented by its associated proteins (nodes) as the edges cannot be interpreted as a unit in this approach. We used protein information files obtained from the STRING database to map preferred gene names to STRING protein identifiers. Overrepresentation analysis-based Gene Ontology (GO) enrichment analysis [5, 6] with biological process ontologies was performed on filtered predicted results of node2vec, struc2vec and cGAN, using clusterProfiler R library [7] version 4.3.1, and N100 network as background for the analysis. In overrepresentation analysis FDR correction was used to adjust p-values for multiple testing and the significance level was set to 0.05. We separately compared GO term sets enriched in node2vec and struc2vec with the GO enrichment results of our method by calculating the semantic similarities between the GO term sets using GOSemSim R library [8] version 2.20.0. For the semantic similarity measurements GO term sets were simplified and were compared by the corresponding functions with their default settings. To store input file paths and parameters for the analysis we used JSON (JavaScript Object Notation) file which was converted into an R object using rjson R library (https://CRAN.R-project.org/package=rjson, version 0.2.20). With VennDiagram R library (https://CRAN.R-project.org/package=VennDiagram, version 1.7.1) we generated Venn diagrams to summarize the comparison results (Supplementary Fig. 3) and to make interpretable the content of these figures we used R libraries RcolorBrewer (https://CRAN.R-project.org/package=RColorBrewer, version 1.1-2) and gridExtra (https://CRAN.R-project.org/package=gridExtra, version 2.3) to color the Venn diagrams and to

add a legend to them. ClusterProfiler and GOSemSim relies on genome-wide annotation packages (OrgDb) maintained by Bioconductor project [9]. In case of the species *Saccharomyces cerevisiae* the inclusion of org.Sc.sgd.db BioConductor annotation data package [10] was necessary due to difficulties with its OrgDb object. In case of *Sus scrofa*, proportionally fewer genes were included into the enrichment sets compared to other species, which could be caused by poorer mapping between GO terms and gene identifiers.

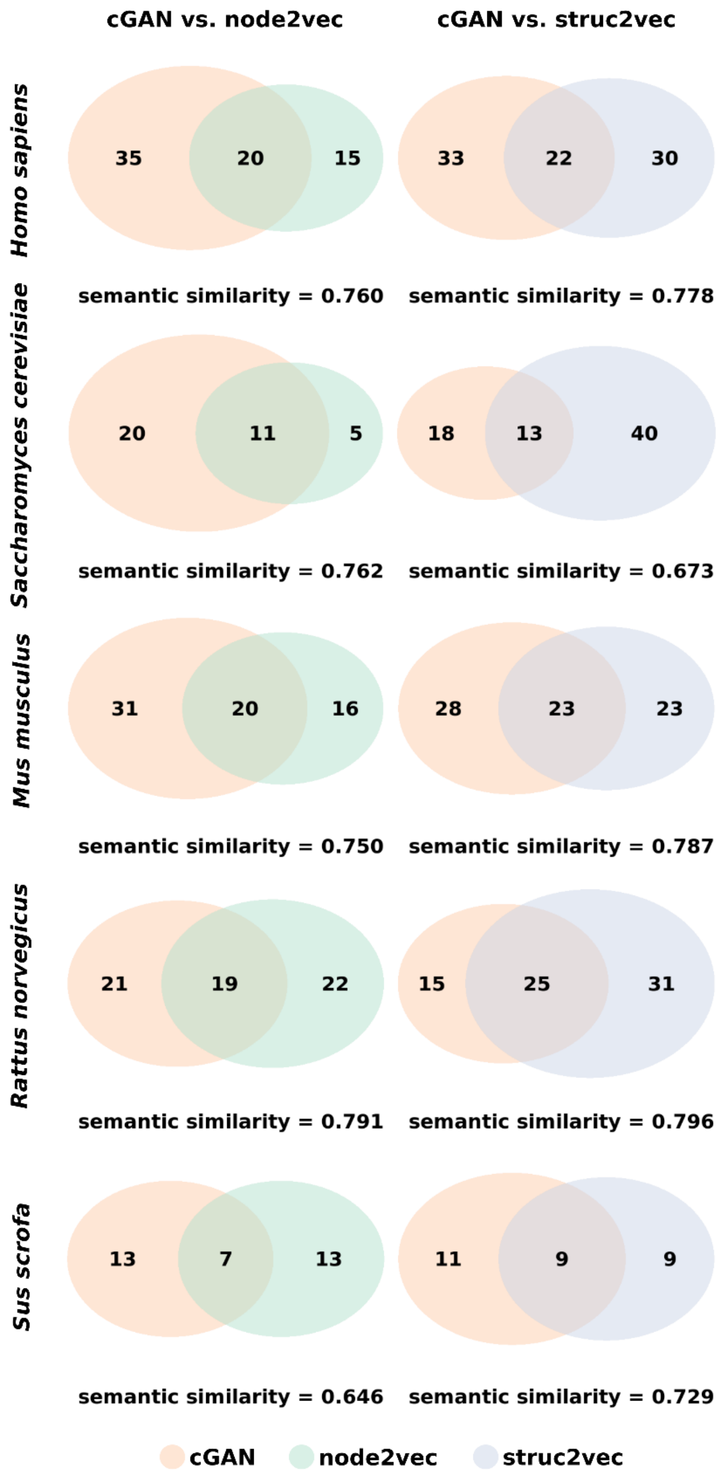

Supplementary Figure 3: Comparison of different prediction methods by enrichment analysis on predicted results from the aspect of biological process ontologies. Venn diagrams of enriched biological process Gene Ontology (GO) terms in our results (cGAN, denoted by pale orange color) in comparison with the results of two prediction methods (node2vec, struc2vec, indicated by pale green and pale blue colors, respectively). With semantic comparisons of GO annotations, we measured the similarities between GO term sets (semantic similarity).

In a similar fashion, we also performed GO enrichment analysis with biological process, molecular function and cellular component ontologies on our results to map certain features of the newly predicted edges. Following the intended use of the proposed cGAN model, for each species we pooled together the top 10% highest confidence interactions from all 10-folds of predictions that were generated during the link prediction from N90 to N100. We separated the true positive and false positive edges to generate the analysis inputs, containing only the nodes connected by the filtered edges as the edges cannot be interpreted as a unit in this approach. Using the corresponding N100 networks as the background for the analysis, we separately compared GO term sets enriched in true positive and false positive sets for each species and each ontology type (Supplementary Table 3, 4) by calculating the semantic similarities between the GO term sets using GOSemSim R library [8]. With VennDiagram R library (https://CRAN.R-project.org/package=rjson, version 1.7.1) we summarized the results of the GO enrichment analysis in Venn diagrams generated separately for true positive and false positive sets and for each ontology type allowing comprehensive overview of the comparison results, presented in Supplementary Fig. 4. R libraries rjson, RColorBrewer, gridExtra, and org.Sc.sgd.db were used as described previously.

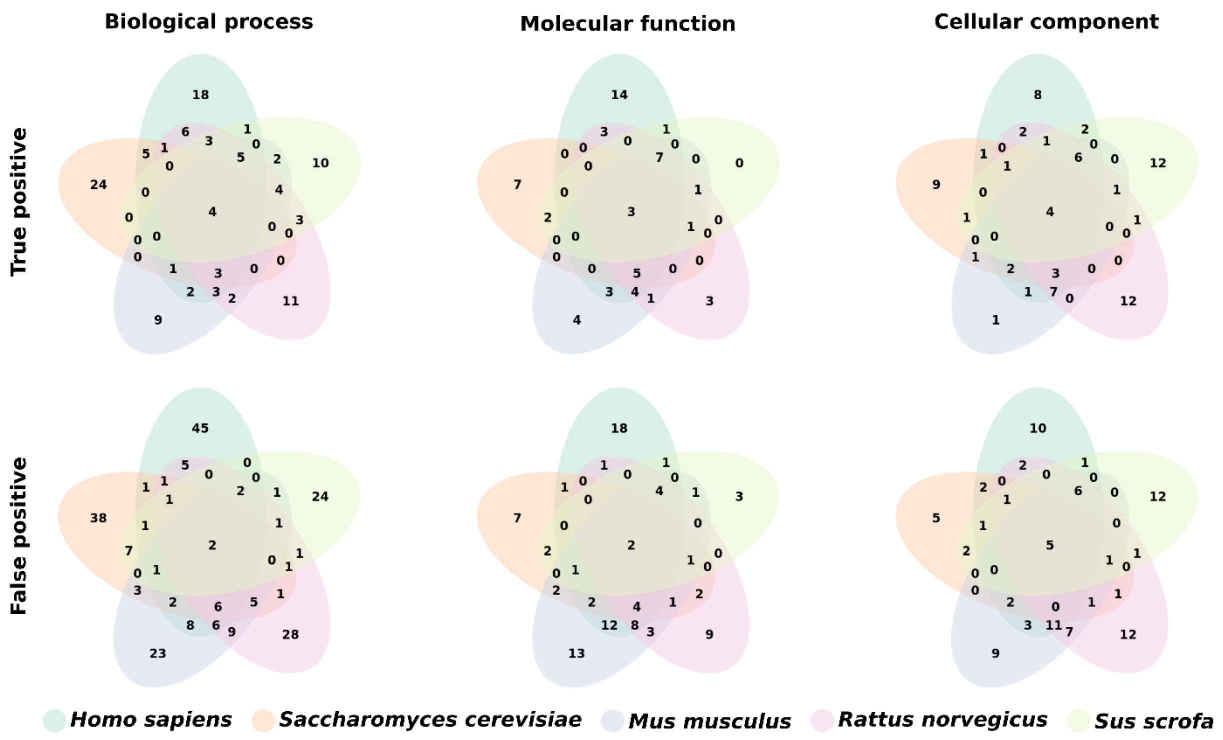

Supplementary Figure 4: Enriched Gene Ontology term set comparisons between true positive and false positive edges derived protein coding gene sets. Venn diagrams visualizing cross-species similarities and differences between Gene Ontology (GO) term sets by ontologies and separately for true positive and false positive sets so the characteristics of true positive and false positive sets are comparable.

Supplementary Table 3: Semantic similarity values of the Gene Ontology terms enriched in true positive and false positive edges derived protein coding gene sets.

Species	Biological process	Molecular function	Cellular component
Homo sapiens	0.759	0.807	0.822
Saccharomyces cerevisiae	0.667	0.745	0.771
Mus musculus	0.666	0.772	0.774
Rattus norvegicus	0.709	0.791	0.826
Sus scrofa	0.653	0.821	0.893

Supplementary Table 4: Gene Ontology terms that enriched in all examined species.

	True positive	False positive
**Biological process**	**GO:0022613** ribonucleoprotein complex biogenesis<br>**GO:0140053** mitochondrial gene expression<br>**GO:0071826** ribonucleoprotein complex subunit organization<br>**GO:1901566** organonitrogen compound biosynthetic process	**GO:0022613** ribonucleoprotein complex biogenesis<br>**GO:0006974** cellular response to DNA damage stimulus
**Molecular function**	**GO:0003735** structural constituent of ribosome<br>**GO:0003899** DNA-directed 5'-3' RNA polymerase activity<br>**GO:0140296** general transcription initiation factor binding	**GO:0003735** structural constituent of ribosome<br>**GO:0140640** catalytic activity, acting on a nucleic acid
**Cellular component**	**GO:0000502** proteasome complex<br>**GO:0000428** DNA-directed RNA polymerase complex<br>**GO:1990904** ribonucleoprotein complex<br>**GO:0120114** Sm-like protein family complex	**GO:0005761** mitochondrial ribosome<br>**GO:0000502** proteasome complex<br>**GO:0120114** Sm-like protein family complex<br>**GO:1990904** ribonucleoprotein complex<br>**GO:0048475** coated membrane

4. Gene and transcript length analysis

For gene and transcript length analysis the protein-coding transcript (coding sequence with untranslated regions) and gene lengths (base pair count from gene start to gene end position) were downloaded from Ensembl via the BioMart data mining web-based tool (*Homo sapiens*: GRCh38.p13, *Saccharomyces cerevisiae*: R64-1-1, *Mus musculus*: GRCm39, *Rattus norvegicus*: mRatBN7.2, *Sus scrofa*: Sscrofa11.1, Ensembl release: 105, access date: December 2021) for all species [11, 12]. We used the same filtering as for the GO analysis of true positive and false positive sets to generate the analysis inputs. The true positive and false positive lengths were statistically compared with Mann-Whitney-Wilcoxon test [13, 14] and based on mean values we determined which set has longer gene or transcript lengths on average (Supplementary Table 5). R library rjson was used as described previously.

Supplementary Table 5: Length analysis results of the true positive and false positive protein coding genes and transcripts.

Species	Length (bp)	True positive	True positive	p-value
Homo sapiens	Gene	42528	65466	< 0.001
	Transcript	2991	3718	< 0.001
Saccharomyces cerevisiae	Gene	1362	1629	< 0.001
	Transcript	1298	1598	< 0.001
Mus musculus	Gene	31088	48241	< 0.001
	Transcript	2742	3369	< 0.001
Rattus norvegicus	Gene	27785	48435	< 0.001
	Transcript	2203	2692	< 0.001
Sus scrofa	Gene	58806	70645	0.005
	Transcript	3200	3465	0.003

References

1. Grover A, Leskovec J. node2vec: Scalable Feature Learning for Networks. Proc ACM SIGKDD Int Conf Knowl Discov Data Min. 2016;13-17-August-2016:855–64. http://arxiv.org/abs/1607.00653. Accessed 6 May 2021.

2. Leskovec J, Sosič R. SNAP: A general-purpose network analysis and graph-mining library. ACM Trans Intell Syst Technol. 2016;8. doi:10.1145/2898361.

3. Yue X, Wang Z, Huang J, Parthasarathy S, Moosavinasab S, Huang Y, et al. Graph embedding on biomedical networks: Methods, applications and evaluations. Bioinformatics. 2020;36:1241–51. doi:10.1093/bioinformatics/btz718.

4. Jensen LJ, Kuhn M, Stark M, Chaffron S, Creevey C, Muller J, et al. STRING 8 - A global view on proteins and their functional interactions in 630 organisms. Nucleic Acids Res. 2009;37 SUPPL. 1:D412-D416. doi:10.1093/nar/gkn760.

5. Ashburner M, Ball CA, Blake JA, Botstein D, Butler H, Cherry JM, et al. Gene ontology: tool for the unification of biology. The Gene Ontology Consortium. Nat Genet. 2000;25:25–9. doi:10.1038/75556.

6. Carbon S, Douglass E, Good BM, Unni DR, Harris NL, Mungall CJ, et al. The Gene Ontology resource: enriching a GOld mine. Nucleic Acids Res. 2021;49:D325–34. doi:10.1093/NAR/GKAA1113.

7. Yu G, Wang LG, Han Y, He QY. clusterProfiler: an R package for comparing biological themes among gene clusters. OMICS. 2012;16:284–7. doi:10.1089/OMI.2011.0118.

8. Yu G, Li F, Qin Y, Bo X, Wu Y, Wang S. GOSemSim: an R package for measuring semantic similarity among GO terms and gene products. Bioinformatics. 2010;26:976–8. doi:10.1093/BIOINFORMATICS/BTQ064.

9. Gentleman RC, Carey VJ, Bates DM, Bolstad B, Dettling M, Dudoit S, et al. Bioconductor: open

software development for computational biology and bioinformatics. Genome Biol. 2004;5:R80. doi:10.1186/GB-2004-5-10-R80/FIGURES/3.

10. M C. org.Sc.sgd.db: Genome wide annotation for Yeast. R package version 3.14.0. 2019.

11. Howe KL, Achuthan P, Allen J, Allen J, Alvarez-Jarreta J, Ridwan Amode M, et al. Ensembl 2021. Nucleic Acids Res. 2021;49:D884–91. doi:10.1093/NAR/GKAA942.

12. Kinsella RJ, Kähäri A, Haider S, Zamora J, Proctor G, Spudich G, et al. Ensembl BioMarts: a hub for data retrieval across taxonomic space. Database (Oxford). 2011;2011. doi:10.1093/DATABASE/BAR030.

13. Neuhäuser M. Wilcoxon–Mann–Whitney Test. Int Encycl Stat Sci. 2011;:1656–8. doi:10.1007/978-3-642-04898-2_615.

14. Kruskal WH. Historical Notes on the Wilcoxon Unpaired Two-Sample Test. J Am Stat Assoc. 1957;52:356–60.
